# Schwarz alternating methods for anisotropic problems with prolate spheroid boundaries

**DOI:** 10.1186/s40064-016-3063-y

**Published:** 2016-08-26

**Authors:** Zhenlong Dai, Qikui Du, Baoqing Liu

**Affiliations:** 1 Jiangsu Key Laboratory for NSLSCS, School of Mathematics Sciences, Nanjing Normal University, No. 1 Wenyuan Road, Nanjing, 210023 People’s Republic of China; 2 School of Applied Mathematics, Nanjing University of Finance and Economics, No. 3 Wenyuan Road, Nanjing, 210023 People’s Republic of China

**Keywords:** Schwarz alternating algorithm, Exterior anisotropic problem, Prolate ellipsoidal, Artificial boundary, Iteration method

## Abstract

The Schwarz alternating algorithm, which is based on natural boundary element method, is constructed for solving the exterior anisotropic problem in the three-dimension domain. The anisotropic problem is transformed into harmonic problem by using the coordinate transformation. Correspondingly, the algorithm is also changed. Continually, we analysis the convergence and the error estimate of the algorithm. Meanwhile, we give the contraction factor for the convergence. Finally, some numerical examples are computed to show the efficiency of this algorithm.

## Background

How to deal with boundary value problems has always been a essential part of partial differential equation. Finite difference method (FDM) (Evans 1977) and finite element method (FEM) (Brenner and Scott 1996) are the most widely used method to solve PDE numerically. These two methods become in vain when it comes to the problem over unbounded domain. To overcome this, boundary element method (BEM), which can reduce the dimension of the computational domain and is suitable for solving problems in the unbounded domains, is proposed in Feng (1980). Although, it is better to handle BEM with infinite regions, it doesn’t work so well as FEM in bounded ones. Hence, the coupling of BEM and FEM becomes the interest of researchers. Papers MacCamy and Marin (1980), Hsiao and Porter (1986), Wendland (1986), Costabel (1987), Han (1990) had focused on this method. In 1983, Feng firstly proposed a direct and natural coupling method. Later in the same year, Feng and Yu (1983) formally named the method as natural boundary element method (NBEM). Meanwhile, the DtN method, which has the similar principle with NBEM, is proposed in Keller and Givoli (1989), Grote and Keller (1995). Du and Yu (2001), Hu and Yu (2001), Gatica et al. (2003), Koyama (2007), Koyama (2009), Das and Mehrmann (2016), Das and Natesan (2014), Das (2015) and references therein present the applications of this methods.

Among the reasons that effects the NBEM, the shape of artificial boundary is the essential one. Classically, circle (Givoli and Keller 1989) and spherical (Grote and Keller 1995; Wu and Yu 1998, 2000a) are chosen as the artificial boundaries. Few papers Grote and Keller (1995), Wu and Yu (2000b), Huang and Yu (2006) focus on the special artificial boundaries. These papers also proved the classic artificial boundaries were not suitable for the problem with irregular shape. On the other hand, the coupling of FEM and BEM are not enough as the performance of computer developed. The domain decomposition method (DDM) (Brenner and Scott 1996), which separates the infinite region as sum of bounded one and unbounded one with an artificial boundary on which an iteration method is constructed in, is applied on the NBEM (Yu 1994). Wu and Yu (2000b) applied this method over an infinite region. Continually, Huang et al. (2009) and Luo et al. (2013) applied this method in different problems.

In this paper, we consider the anisotropic harmonic problem over an exterior three-dimensional domain. A Schwartz alternating method is designed for the numerical solution with prolate artificial boundaries.

The outline of the paper is as follows. In “Schwarz alternating algorithm based on NBR” section, we divide the original domain $\Omega$ into two overlapping subdomains $\Omega _1$ and $\Omega _2$ by choosing two artificial boundaries $\Gamma _1$ and $\Gamma _2$, then we construct the Schwarz alternating algorithm. We prove the convergence of the algorithm in “Convergence of the algorithm” section. The convergence rate of the algorithm is analysed in the “Analysis of the convergence rate” section. In “The error estimates of the algorithm” section, we deduce the error estimates of the discrete algorithm. In “Numerical results” section, numerical examples are computed to express the advantages of this method. Finally, we give some conclusions in “Conclusions” section.

## Schwarz alternating algorithm based on NBR

Let $\Omega \subset R^{3}$ be a cuboid Lipschitz unbounded domain and $\Gamma _{0}=\partial \Omega$ is its boundary. We consider the following exterior Dirichlet problem

1 $$\begin{aligned} \left\{ \begin{array}{ll} \displaystyle -\left( K_{1}\frac{\partial ^{2}}{\partial {x^{2}}}+K_{1}\frac{\partial ^{2}}{\partial {y^{2}}}+ K_{2}\frac{\partial ^{2}}{\partial {z^{2}}}\right) u=0, &{}\quad {\hbox {in}}\, \Omega , \\ u=g, &{}\quad {\hbox {on}}\,\Gamma _0, \\ u \rightarrow 0 &{}\quad {\hbox {as}}\, r\rightarrow \infty , \end{array} \right. \end{aligned}$$

where $K_{1}$ and $K_{2}$ are two different anisotropic parameters, *g* is a given function that satisfies $g\in H^{1/2}(\Gamma _0)$, and $r=\sqrt{x^2+y^2+z^2}$. The third item of Eq. (1) keeps the existence and uniqueness of the solution.

**Fig. 1:**
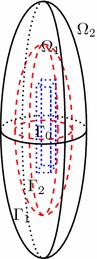
Domain participation

Let $\Gamma _1=\{(x,y,z): \frac{x^2+y^2}{d^2}+\frac{z^2}{c^2}=1,\ c>d>0\}$ and $\Gamma _2=\{(x,y,z): \frac{x^2+y^2}{b^2}+\frac{z^2}{a^2}=1,\ a>b>0\}$ denote two artificial prolate spheroids. For clarity, we must mention that $d>b$ and $c>a.$ This means that $\Gamma _2$ is totally inside $\Gamma _1$. Define $\Omega _2$ as the unbounded domain outside the boundary $\Gamma _2$ and $\Omega _1$ be a bounded domain between $\Gamma _0$ and $\Gamma _1$ (see Fig. 1).

According to DDM (Brenner and Scott 1996), we construct the Schwarz alternating method as follows:

2 $$\begin{aligned} \left\{ \begin{array}{ll} \displaystyle -\left( K_{1}\frac{\partial ^{2}}{\partial {x^{2}}}+K_{1}\frac{\partial ^{2}}{\partial {y^{2}}} +K_{2}\frac{\partial ^{2}}{\partial {z^{2}}}\right) u^{(2k+1)}_{1}=0, &{}\quad {\hbox {in}} \,\Omega _1, \\ u^{(2k+1)}_{1}=u^{(2k)}_{2}, &{}\quad {\hbox {on}} \,\Gamma _1,\\ u^{(2k+1)}_{1}=g, &{}\quad {\hbox {on}}\,\Gamma _0, \end{array} \right. \end{aligned}$$

and

3 $$\begin{aligned} \left\{ \begin{array}{ll} \displaystyle -\left(K_{1}\frac{\partial ^{2}}{\partial {x^{2}}}+K_{1}\frac{\partial ^{2}}{\partial {y^{2}}} +K_{2}\frac{\partial ^{2}}{\partial {z^{2}}}\right) u^{(2k+2)}_{2}=0, &\quad {\hbox{in}}\;\Omega _2, \\ u^{(2k+2)}_{2}=u^{(2k+1)}_{1}, &\quad {\hbox{on}}\;\Gamma _2, \\ u^{(2k+2)}_{2} \rightarrow 0, &\quad {\hbox {as}}\; r \rightarrow \infty , \end{array} \right. \end{aligned}$$

where $$k=0,1,\ldots $$ and $$u_2^{(0)}=\widetilde{u}$$.

Setting the initial value of function $$u_2^{(0)}$$ on boundary $$\Gamma _1$$ as $$u_2^{(0)}|_{\Gamma _1}=0$$. Hence, we can solve the problem (2). Furthermore, with the limitation of $$u_1^{(1)}$$ on $$\Gamma _2$$, one solves the problem (3). Sequentially, we solve the problem in $$\Omega _1$$ again with substituting the value of solution $$u_2^{(2)} $$ on $$\Gamma _1$$. Then , we repeat the steps for $$k=1,2,\ldots $$ and so on.

By the above description, obviously, we applied FEM in the problem over $\Omega _1$ and BEM (Feng and Yu 1983) in $\Omega _2$. Before using BEM to solve problem (3), the following transformation is introduced.

4 $$\begin{aligned} \left\{ \begin{array}{l} x=\sqrt{K_1} {x_1}, \\ y=\sqrt{K_1} {y_1}, \\ z=\sqrt{K_2} {z_1}. \end{array} \right. \end{aligned}$$

For simplicity, the corresponding signals under the coordinate system $(x_1, y_1, z_1)$ can be defined by adding an apostrophe on the original ones, e.g. $\Omega \rightarrow \Omega'$. Therefore, problem (3) can be expressed as the harmonic problem according to the new coordinate system.

5 $$\begin{aligned} \left\{ \begin{array}{ll} \displaystyle -\left(\frac{\partial ^{2}}{\partial {x_{1}^{2}}}+\frac{\partial ^{2}}{\partial {y_{1}^{2}}} +\frac{\partial ^{2}}{\partial {z_{1}^{2}}}\right) u^{(2k+2)}_{2}=0, &{}\quad {\hbox{in}}\,\Omega'_{2}, \\ u^{(2k+2)}_{2}=u^{(2k+1)}_{1}, &{}\quad {\hbox{on}}\,\Gamma '_2, \\ u^{(2k+2)}_{2} \rightarrow 0, &{}\quad {\hbox {as}}\,r' \rightarrow \infty , \end{array} \right. \end{aligned}$$

We introduce the prolate spheroidal coordinates $(\mu ,\theta ,\varphi )$, such that $\Gamma' _2$ coincides with the prolate spheroid $\mu =\mu _2$ and $\Omega'_2=\{(\mu ,\theta ,\varphi )| \mu> \mu _2 >0, \theta \in [0,\pi ], \varphi \in [0,2\pi ]\}$.

6 $$\begin{aligned} \left\{ \begin{array}{ll} x_1=f \sinh \mu \sin \theta \cos \varphi , &{}\quad \mu \ge \mu _2 >0, \\ y_1=f \sinh \mu \sin \theta \sin \varphi , &{}\quad \theta \in [0,\pi ], \\ z_1=f \cosh \mu \cos \theta , &{}\quad \varphi \in [0,2\pi ], \end{array} \right. \end{aligned}$$

where $$f=\sqrt{\frac{a^2}{K_2}-\frac{b^2}{K_1}}$$, $$a=f \cosh \mu _2$$ and $$b=f \sinh \mu _2$$.

For simplicity, the problem (5) can be expressed as

7 $$\begin{aligned} \left\{ \begin{array}{ll} -\Delta u=0, &{}\quad {\hbox {in}}\, \Omega'_2, \\ u=u_{1}, &{}\quad {\hbox {on}}\, \Gamma'_2, \\ u \rightarrow 0, &{}\quad {\hbox {as}}\,r' \rightarrow \infty . \\ \end{array} \right. \end{aligned}$$

By the separation of variable (Zhang and Jin 1996), we have the solution of (7) as follows

8 $$\begin{aligned} u(\mu ,\theta ,\varphi ) &= \sum _{n=0}^{\infty }\sum _{m=-n}^{n}\frac{Q^m_n(\cosh \mu )}{Q^m_n(\cosh \mu _2)}U_{nm}Y_{nm}(\theta ,\varphi ) \\&\equiv  H(u_2,\mu ,\theta ,\varphi ),\quad \mu \ge \mu _2>0, \end{aligned}$$

where

$$\begin{aligned} U_{nm}&= \int _0^{2\pi }\int _0^\pi u_2(\mu _2,\theta ,\varphi )Y^*_{nm}(\theta ,\varphi )\sin (\theta )d\theta d\varphi ,\\ Y^*_{nm}&= (-1)^mY_{nm}(\theta ,\varphi )=(-1)^m\sqrt{\frac{2n+1}{4\pi } \frac{(n-m)!}{(n+m)!}}P^m_n(\cos (\theta ))e^{im\varphi }. \end{aligned}$$

$P^m_n$ and $Q^m_n$ are the first and second kind of the associated Legendre functions. Therefore, the solution *u* of (7) restricted on $\Gamma'_1$ can be expressed as

$$ u(\mu _1,\theta ,\varphi )=H(u_2,\mu _1,\theta ,\varphi ). $$

Similarly, we have the equivalent problem of (2). Thus, the Schwarz alternating algorithm can be expressed as follows:

9 $$\begin{aligned} \left\{ \begin{array}{ll} -\Delta u_1^{(2k+1)}=0, &{}\quad {\hbox {in}}\,\Omega'_1, \\ u_1^{(2k+1)}=g', &{}\quad {\hbox {on }}\,\Gamma'_0, \\ u_1^{(2k+1)}=u_2^{(2k)}, &{}\quad {\hbox {on}}\,\Gamma'_1, \end{array} \right. \end{aligned}$$

and

10 $$\begin{aligned} \left\{ \begin{array}{ll} -\Delta u_2^{(2k+2)}=0, &{}\quad {\hbox {in}}\,\Omega'_2, \\ u_2^{(2k+2)}=u_1^{(2k+1)}, &{}\quad {\hbox {on}}\,\Gamma'_2,\\ u_2^{(2k+2)} \rightarrow 0, &{}\quad {\hbox {as}}\,r' \rightarrow \infty . \\ \end{array} \right. \end{aligned}$$

where $k=0,1,\ldots$. The detail is similar to the original.

## Convergence of the algorithm

We define the following spaces

$$\begin{aligned}&W^1_0(\Omega') = \left\{ v\left| \frac{v}{\sqrt{1+x_1^2+y_1^2+z_1^2}}\in L^2(\Omega')\right. ;\; \frac{\partial v}{\partial x_1},\frac{\partial v}{\partial y_1},\frac{\partial v}{\partial z_1}\in L^2(\Omega') \right\} ,\\&{\mathring{W}}_0^1(\Omega')=\{v\in W_0^1(\Omega')|v|_{\Gamma'_0}=0\}. \end{aligned}$$

Solutions of (9) and (10) are in $V_1=H_0^1(\Omega'_1)$ and $V_2={\mathring{W}}_0^1(\Omega'_2)$, respectively. Moreover, we denote the ${\mathring{W}}_0^1(\Omega')$ as *V*. Both functions of $V_1$ and $V_2$ can be extended into *V*. For example, we can extend $u \in V_1$ by zero in $\Omega' {\setminus} \Omega'_1$ to a function in *V*.

Hence, we have the equivalent variational form of (5):

11 $$\begin{aligned} \left\{ \begin{array}{ll} \text {Find}\, w=u-\widetilde{u} \in {{\mathring{W}}^1_0(\Omega')},&\quad \text {such that}\\ D_{\Omega'}(w,v)=-D_{\Omega'}(\widetilde{u},v), &\quad \forall v \in {{\mathring{W}}^1_0(\Omega')}, \end{array} \right. \end{aligned}$$

where $D_{\Omega'}(u,v)=\int _{\Omega'}\nabla u\cdot \nabla v \text {d} x_1\text {d} y_1\text {d}z_1$, $\widetilde{u}\in W^1_0(\Omega')$ has compact support and $\widetilde{u}|_{\Gamma'_0}=g$. $|u|_1=\sqrt{D_{\Omega'}(u,u)}$ is an equivalent norm of ${\mathring{W}}_0^1(\Omega')$. If $g\in H^{\frac{1}{2}}(\Gamma'_0)$, then there exists $\widetilde{u}$ such that the solution of (11) exists and is uniquely determined.

Then (9) and (10) are equivalent to the following variational problems:

12 $$\begin{aligned} \left\{ \begin{array}{ll} {\hbox {Find}}\, w_1^{(2k+1)}=u_1^{(2k+1)}-u^{(2k)}|_{\Omega'_1}\in V_1, &\quad {\hbox {such that}}\\ D_{\Omega'_1}(w_1^{(2k+1)},v)=-D_{\Omega'_1}(u^{(2k)},v), &\quad \forall v\in V_1,\end{array} \right. \end{aligned}$$

and

13 $$\begin{aligned} \left\{ \begin{array}{ll} \text {Find}\, w_2^{(2k+2)}=u_2^{(2k+2)}-u^{(2k+1)}|_{\Omega'_2}\in V_2,& \quad {\hbox {such that}}\\ D_{\Omega'_2}(w_2^{(2k+2)},v)=-D_{\Omega'_2}(u^{(2k+1)},v),& \quad \forall v\in V_2.\end{array} \right. \end{aligned}$$

Let

$$\begin{aligned} u^{(2k+1)}&= \left\{ \begin{array}{ll} u_1^{(2k+1)}, &\quad \text {in}\ \Omega'_1\\ u_2^{(2k)},&{}\text {in}\ \Omega'\backslash \Omega'_1,\end{array}\right. \\ u^{(2k+2)}&= \left\{ \begin{array}{ll} u_1^{(2k+1)}, &\quad \text {in}\ \Omega'\backslash \Omega'_2\\ u_2^{(2k+2)},&{}\text {in}\ \Omega'_2,\end{array}\right. \end{aligned}$$

and $u^{(0)}=\widetilde{u},$ then we have

$$\begin{aligned} D_{\Omega'}(u-u^{(2k+1)},v_1)&= 0,\quad \forall v_1\in V_1,\\ D_{\Omega'}(u-u^{(2k+2)},v_2)&= 0,\quad \forall v_2\in V_2. \end{aligned}$$

Noticing

$$ u^{(2k+1)}-u^{(2k)}\in V_1,\quad u^{(2k+2)}-u^{(2k+1)}\in V_2 $$

and

$$ u-u^{(2k+1)}\in V,\quad u-u^{(2k+2)}\in V,$$

Hence,

14 $$ u^{(2k+1)}-u^{(2k)}=P_{V_1}(u-u^{(2k)}),\quad u^{(2k+2)}-u^{(2k+1)}=P_{V_2}\left(u-u^{(2k+1)}\right)$$

where $P_{V_i}{:}V\rightarrow V_i \;(i=1,2)$ means the projection operator under the inner product $D_{\Omega'}(\cdot ,\cdot )$ in *V*. Thus (14) is equivalent to

15 $$ \left\{ \begin{array}{l} u-u^{(2k+1)}=P_{V^\bot _1}(u-u^{(2k)}),\\ u-u^{(2k+2)}=P_{V^\bot _2}(u-u^{(2k+1)}).\end{array} \right. $$

Denote the errors as $e^{(k)}_{i}=u-u^{(k)} (i=1,2)$. This leads to

$$ \left\{ \begin{array}{l} e^{(2k+1)}_1=P_{V^\bot _1}P_{V^\bot _2}e_1^{(2k-1)},\\ e^{(2k+2)}_2=P_{V^\bot _2}P_{V^\bot _1}e_2^{(2k)},\end{array} \right.$$

This implies that, if $\{e_1^{(2k+1)}\}$ and $\{e_2^{(2k)}\}$ are convergent, then their limits are in $V_1^\bot \cap V_2^\bot$. Similar to the proofs given in Yu (1994, 2002); Luo et al. (2013) we can show the following result.

### **Theorem 1**

*There exists a constant*$\alpha$, $0\le \alpha <1$, *such that*

$$ \left\Vert e_1^{(2k+1)}\right\Vert _1\le \alpha ^{2k}\left\Vert e_1^{(1)}\right\Vert _1,\quad \left\Vert e_2^{(2k+2)}\right\Vert _1\le \alpha ^{2k+2}\left\Vert e_2^{(0)}\right\Vert _1. $$

It is obvious to conclude $\alpha$ keeps the convergence of Schwarz alternating method. In the next section, we will prove the contraction factor $\alpha$.

## Analysis of the convergence rate

By Theorem 1, one may find the convergence rate of the above Schwarz alternating algorithm is closely related to the contraction factor $\alpha$, i.e. the overlapping extent of $\Omega'_1$ and $\Omega'_2$. Although it can be deduced intuitively that the larger the overlapping part is, the faster convergence rate will be, yet we find it difficult to analyse the convergence rate for general unbounded domain $\Omega'$. However, under certain assumptions, we can find out the relationship between contraction factor $\alpha$ and overlapping extent of $\Omega'_1$ and $\Omega'_2$. We define three prolate spheroids with the same semi-interfocal distance

16 $$ \Gamma'_i=\{(\mu ,\theta ,\varphi ): \mu =\mu _i,\theta \in [0,\pi ],\varphi \in [0,2\pi ]\},\quad i=0,1,2, $$

where $$\mu _1>\mu _2>\mu _0>0$$.

We consider the following boundary value problem over domain $\Omega'_1$

17 $$ \left\{ \begin{array}{ll} -\Delta u=0, &{}\quad {\hbox {in}}\,\Omega'_1,\\ u=g_0, &{}\quad {\hbox {on}}\,\Gamma'_0, \\ u=g_1, &{}\quad {\hbox {on}}\,\Gamma'_1. \end{array} \right.$$

Suppose that

18 $$ g_i(\theta ,\varphi )=\sum ^{+\infty }_{n=0}\sum ^{n}_{m=-n}G^{(i)}_{nm}Y_{nm}(\theta ,\varphi ), \quad i=0,1, $$

where

$$ G^{(i)}_{nm}=\int _0^\pi \int _0^{2\pi } g_i(\theta ,\varphi )Y^*_{nm}(\theta ,\varphi )\sin (\theta )d\theta d\varphi , \quad i=0,1. $$

Then by the separation of variables, we can obtain the solution of (17)

19 $$\begin{aligned} u(\mu ,\theta ,\varphi )=\sum ^{+\infty }_{n=0}\sum ^{n}_{m=-n} \frac{\left( S(\mu ,\mu _1)G_{nm}^{(0)}+S(\mu _0,\mu )G_{nm}^{(1)}\right) }{S(\mu _0,\mu _1)}Y_{nm}(\theta ,\varphi ), \end{aligned}$$

where $S(x,y)=P_n^m(\cosh x)Q_n^m(\cosh y)-P_n^m(\cosh y)Q_n^m(\cosh x)$. According to the property of the associated Legendre functions (Gradshteyn and Kyzhik 1980), we have the following lama.

### **Lemma 1**

*Let*

$$ P_{n}^{m}(x)=\frac{d^{n+m}}{dx^{n+m}}(x^2-1)^n,$$

*wheren*, *mare both nonnegative integers. If*$0\le m<n$, *then*$P_{nm}(x)$*has*$n-m$*different zeros*$-1 = \alpha _1\le \alpha _2\le \cdots \le \alpha _{n-m} = 1$*with*$\alpha _i=-\alpha _{n-m-(i-1)}, \quad i=1,\ldots ,n-m-1.$

### **Lemma 2**

*If*$\mu >\mu _0$, *then we conclude*

20 $$ \frac{P_n^m(\cosh \mu _0)}{P_n^m(\cosh \mu )}<\left( \frac{\cosh \mu _0}{\cosh \mu }\right) ^n, $$

*and*

21 $$ \frac{Q_n^m(\cosh \mu )}{Q_n^m(\cosh \mu _0)}<\left( \frac{\cosh \mu _0}{\cosh \mu }\right) ^n. $$

### *Proof*

By the definition of $P^m_n(x)$ we have

$$ \frac{P_n^m(\cosh \mu _0)}{P_n^m(\cosh \mu )}=\left( \frac{\sinh \mu _0}{\sinh \mu }\right) ^{m-2} \frac{\prod \limits ^{n-m}_{i=1}(\cosh \mu _0-\alpha _i)}{\prod \limits ^{n-m}_{i=1}(\cosh \mu -\alpha _i)}. $$

For monotonicity, the following holds for $i=1,2,\dots ,n-m,$

$$ \frac{(\cosh \mu _0-\alpha _i)(\cosh \mu _0-\alpha _{n-m-i+1})}{(\cosh \mu -\alpha _i)(\cosh \mu -\alpha _{n-m-i+1})} =\frac{(\cosh ^2\mu _0-\alpha _i^2)}{(\cosh ^2\mu -\alpha _i^2)}<\frac{\cosh ^2\mu _0}{\cosh ^2\mu }. $$

Hence,

$$ \frac{P_n^m(\cosh \mu _0)}{P_n^m(\cosh \mu )}<\left( \frac{\cosh \mu _0}{\cosh \mu }\right) ^n. $$

$$\square $$

On the other hand, (21) can be easily proved by the proposition of Huang and Yu (2006),

### **Theorem 2**

*Suppose*$g_0$*is continuous on*$\Gamma _0$*and* (16) *holds. If we apply the Schwarz alternating algorithm given in * “*Schwarz alternating algorithm based on NBR*”*section, then*

22 $$ \sup _{\overline{\Omega }_1}|u-u^{(2k+1)}|\le C_1\alpha ^{k} $$

*and*

23 $$ \sup _{\overline{\Omega }_2}|u-u^{(2k+2)}|\le C_2\alpha ^{k+1} $$

*hold true, the constant*$$C_i$$$$(i=1,2)$$*depend only on*$$g_0$$*and*$$\displaystyle \frac{Q^m_n(\cosh \mu _i)}{Q^m_n(\cosh \mu _0)}$$*while*

24 $$0<\alpha =\frac{Q_n^m(\cosh \mu _1)S(\mu _0,\mu _2)}{Q_n^m(\cosh \mu _2)S(\mu _0,\mu _1)}<1. $$

### *Proof*

Similar to (8), so the solution of the unbounded problem outside of $\Gamma _0$ can be expressed as

$$ u(\mu ,\theta ,\varphi )=\sum _{n=0}^{\infty }\sum _{m=-n}^{n}\frac{Q^m_n(\cosh \mu )}{Q^m_n(\cosh \mu _0)}G^{(0)}_{nm}Y_{nm}(\theta ,\varphi ), \quad\mu \ge \mu _0. $$

Let $$\tilde{u}=0$$.

By using the algorithm, one has

$$\begin{aligned}&u(\mu ,\theta ,\varphi )-u^{(2k+1)}(\mu ,\theta ,\varphi )\\&\qquad =\displaystyle {\sum ^{+\infty }_{n=0}\sum ^{n}_{m=-n}}\frac{Q^m_n(\cosh \mu _1)}{Q^m_n(\cosh \mu _0)} \left[ \frac{Q^m_n(\cosh \mu _1)S(\mu _0,\mu _2)}{Q^m_n(\cosh \mu _2)S(\mu _0,\mu _1)}\right] ^{k} \frac{S(\mu _0,\mu )}{S(\mu _0,\mu _1)}G^{(0)}_{nm}Y_{nm}(\theta ,\varphi ), \end{aligned}$$

where $$\mu _0\le \mu \le \mu _1.$$$$\square $$

By defining

$$ \alpha =\frac{Q_n^m(\cosh \mu _1)S(\mu _0,\mu _2)}{Q_n^m(\cosh \mu _2)S(\mu _0,\mu _1)}, $$

we will show (24).

From Lemma 2, we have

$$ T(\mu )>\left( \frac{\cosh \mu }{\cosh \mu _0}\right) ^{2n}>1,\quad \mu >\mu _0, $$

and

$$ \frac{T(\mu _1)}{T(\mu _2)}>\left( \frac{\cosh \mu _1}{\cosh \mu _2}\right) ^{2n}>1, $$

where $T(\mu )$ is defined as

$$ T(\mu )=\frac{P_n^m(\cosh \mu )Q_n^m(\cosh \mu _0)}{P_n^m(\cosh \mu _0)Q_n^m(\cosh \mu )}. $$

Since

$$ \alpha =\frac{T(\mu _2)-1}{T(\mu _1)-1}=1+\frac{T(\mu _2)-T(\mu _1)}{T(\mu _1)-1}, $$

we obtain $0<\alpha <1.$ Hence, (22) is accomplished.

Obviously, (23) can be proved with similar process. Finally, the theorem is proved.

### *Remark*

 The convergence is related on the overlapping part of $\Omega'_1$ and $\Omega'_2$. From Theorem 2, we conclude the larger the overlapping part is, the smaller the contraction factor $\alpha$ will be, which identically means the faster the Schwarz alternating algorithm converging.

## The error estimates of the algorithm

Denote $S_h(\Omega'_1)$ as the linear finite element space over $\Omega'_1$, where the elements are partitioned as tetrahedrons. Let

$$ {\mathring{S}}_h(\Omega'_1)=\left\{ v_h\in S_h(\Omega'_1)|v_h|_{\Gamma '_0\cup \Gamma '_1}=0\right\}.$$

${\mathring{S}}_h(\Omega'_1)$ can be regarded as the subspace of *V* by zero extension. Therefore, we have the discrete Schwarz alternating algorithm as

25 $$\begin{aligned} \left\{ \begin{array}{ll} {\hbox {Find}}\, w_{1h}^{(2k+1)}=u_{1h}^{(2k+1)}-u_h^{(2k)}|_{\Omega'_1}\in {\mathring{S}}_h(\Omega'_1) &\quad {\hbox {such that}}\\ D_{\Omega'_1}(w_{1h}^{(2k+1)},v_h)=-D_{\Omega'_1}(u_h^{(2k)},v_h), &\quad \forall v_h\in {\mathring{S}}_h(\Omega'_1),\end{array} \right. \end{aligned}$$

and

26 $$\begin{aligned} \left\{ \begin{array}{l} {\hbox {Find}} w_{2h}^{(2k+2)}=u_{2h}^{(2k+2)}-u_h^{(2k+1)}|_{\Omega'_2}\in V_2 \quad  \text {such that}\\ D_{\Omega'_2}(w_{2h}^{(2k+2)},v)=-D_{\Omega'_2}(u_h^{(2k+1)},v), \quad \forall v_h\in V_2,\end{array} \right. \end{aligned}$$

where

$$\begin{aligned} u_h^{(2k+1)}=\left\{ \begin{array}{ll} u_{1h}^{(2k+1)}, &{}\quad {\hbox {in}}\, \Omega'_1\\ u_h^{(2k)},&{}\quad {\hbox {in}}\, \Omega'\backslash \Omega'_1,\end{array}\right. \\ u_h^{(2k+2)}=\left\{ \begin{array}{ll} u_h^{(2k+1)}, &{} {\hbox {in}}\, \Omega'\backslash \Omega'_2\\ u_{2h}^{(2k+2)},&{}{\hbox {in}}\, \Omega'_2,\end{array}\right. \\ \end{aligned}$$

and $$u_h^{(0)}=\widetilde{u}.$$

By Yu (2002), the solution of (26) can be written as

27 $$ u_{2h}^{(2k+2)}=P\gamma u_h^{(2k+1)}, $$

where $P{:}H^{\frac{1}{2}}(\Gamma _2')\rightarrow W_0^1(\Omega _2')$ denotes Poisson integral operator and $\gamma {:}H^1(\Omega _1')\rightarrow H^{\frac{1}{2}}(\Gamma _2')$ denotes trace operator. Combining with (27) and the discrete algorithm, one can easily have the following iteration value:

$$\begin{aligned} u_h^{(2k+1)}&= \widetilde{u}+ \left\{ \begin{array}{ll} \displaystyle \sum _{i=0}^kw_{1h}^{(2i+1)}, &{}\quad {\hbox {on}}\ \overline{\Omega'}\backslash \Omega'_2\\ \displaystyle \sum _{i=0}^kw_{1h}^{(2i+1)}+\sum _{j=0}^{k-1}\left[ P\gamma w_{1h}^{(2j+1)}-w_{1h}^{(2j+1)}\right] \\ \qquad +\delta _k(P\gamma \widetilde{u}-\widetilde{u}), &{}\quad {\hbox {in}}\ \Omega'_1\backslash (\overline{\Omega'}\backslash \Omega'_2),\\ \displaystyle \sum _{j=0}^{k-1}P\gamma w_{1h}^{(2j+1)}+\delta _k(P\gamma \widetilde{u}-\widetilde{u}), &{}\quad {\hbox {on}}\, \Omega'\backslash \Omega'_1, \end{array} \right. \end{aligned}$$

and

$$\begin{aligned} u_h^{(2k+2)}&= \widetilde{u}+ \left\{ \begin{array}{ll} \displaystyle \sum _{i=0}^kw_{1h}^{(2i+1)}, &{}\quad {\hbox {on}}\, \overline{\Omega'}\backslash \Omega'_2\\ \displaystyle \sum _{i=0}^kw_{1h}^{(2i+1)}+\sum _{j=0}^{k}[P\gamma w_{1h}^{(2j+1)}-w_{1h}^{(2j+1)}]\\ \qquad +(P\gamma \widetilde{u}-\widetilde{u}), &{}\quad {\hbox {in}}\, \Omega'_1\backslash (\overline{\Omega'}\backslash \Omega'_2),\\ \displaystyle \sum _{j=0}^{k-1}P\gamma w_{1h}^{(2j+1)}+(P\gamma \widetilde{u}-\widetilde{u}), &{}\quad {\hbox {on}}\, \Omega'\backslash \Omega'_1, \end{array} \right. \end{aligned}$$

where

$$ \delta _k= \left\{ \begin{array}{ll} 0, &\quad \text {if}\ k=0,\\ 1, &\quad \text {if}\ k>0.\\ \end{array} \right. $$

The term $\sum \nolimits _{j=0}^{k-1}$ vanishes at $k=0$. Set

$$ A_h(\Omega'_2)= \left\{ P\gamma (v_h+\alpha \widetilde{u}+\beta w)-(v_h+\alpha \widetilde{u}+\beta w)|_{\overline{\Omega'}_2}|v_h\in {\mathring{S}}_h(\Omega'_1),\alpha ,\beta \in R,w=u-\widetilde{u} \right\} . $$

Similarly, we have the $A_h(\Omega _2')$ as the subspace of *V*. Hence, $A_h(\Omega'_2)\subset V_2\subset V.$ We have the following variational problem on the discrete space

28 $$\begin{aligned} \left\{ \begin{array}{l} \text {Find}\ v_h^*\in {\mathring{S}}_h(\Omega'_1)+A_h(\Omega'_2)\quad \text {such that}\\ D_{\Omega'}(v_h^*,v_h)=-D_{\Omega'}(\widetilde{u},v_h), \quad \forall v_h\in {\mathring{S}}_h(\Omega'_1)+A_h(\Omega'_2).\end{array} \right. \end{aligned}$$

Obviously, the solution of (28) exists uniquely . Set $u_h^*=v_h^*+\widetilde{u}.$ Similarly in Wu and Yu (2000b), we have the following error estimates.

### **Theorem 3**

*For the discrete Schwarz alternating algorithm and the constant*$\alpha$*inTheorem* 1, *the following error estimates hold*

$$\begin{aligned}&|u-u_h^{(2k+1)}|_1 \le C \,h +\alpha ^{2k}|u_h^*-u_h^{(1)}|_1,\\&|u-u_h^{(2k+2)}|_1 \le C\, h +\alpha ^{2k+2}|u_h^*-u_h^{(0)}|_1. \end{aligned}$$

## Numerical results

Some numerical examples are computed to show the efficiency of our algorithm in this section. Using the method developed in “Schwarz alternating algorithm based on NBR” section. The linear elements is used in the computation of FEM. Computationally, we consider on three meshes: Mesh I, Mesh II and Mesh III. Each mesh is a refinement of its former one, especially as Mesh I is the primary. The refinement is defined as each of elements of the former mesh is divided into eight similar shape equally.

*e* and $e_h$ denote the maximal error of all node functions on $\Gamma _{1h}$, respectively, i.e.,

$$\begin{aligned}&e(k)=\sup _{P_i\in \Omega _{1h}}\left| u(P_i)-u^{(2k+1)}_{1h}(P_i)\right| ,\\&e_h(k)=\sup _{P_i\in \Omega _{1h}}\left| u^{(2k-1)}_{1h}(P_i)-u^{(2k+1)}_{1h}(P_i)\right| . \end{aligned}$$

$q_h(k)$ is the rate of convergence, i.e.

$$ q_h(k)=\frac{e_h(k-1)}{e_h(k)}.$$

Moreover, we use the relative maximum norm ($\Vert E_{u}\Vert _{\infty }$) of the errors between numerical solutions and the exact solutions:

$$ \Vert E_{u}\Vert _{\infty }=\frac{|u-u_h|_{\infty ,\Omega _1}}{|u|_{\infty ,\Omega _1}}. $$

### *Example 1*

Set the cubic $\Omega =\{(x,y,z)| \,|x|\le 1,|y|\le 1,|z|\le 3\}$ and $\Gamma _0$ be its surface of $\Omega$. The exact solution of problem (5) be

$$ u=\frac{x/ \sqrt{K_1}}{((x^2+y^2)/ K_1+ z^2/ K_2)^{3/2}}. $$

Also $$g=u|_{\Gamma _0}$$.

**Fig. 2:**
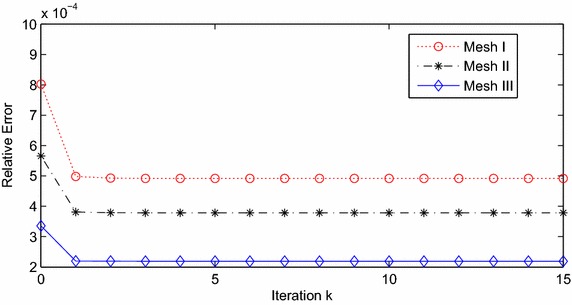
Maximal errors in relative maximum norm

By the theoretical analysis, we take two confocal prolate ellipsoidal surfaces as artificial boundaries, which can be expressed as $\Gamma _1=\{(\mu ,\theta ,\varphi )| \,\mu _1=1.5,\theta \in [0,\pi ],\varphi \in [0,2\pi ]\}$ and $\Gamma _2=\{(\mu ,\theta ,\varphi )| \,\mu _2=1.25,\theta \in [0,\pi ],\varphi \in [0,2\pi ]\}$. And the semi-interfocal distance $f_1=f_2=6$. Moreover, we have $K_1=1$ and $K_2=3$. The efficient results are the case in Tables 1, 2 and Fig. 2.

**Table 1:** The relation between convergence rate and mesh: $\mu _1=1.5$, $\mu _2=1.25$

Mesh	k	Number of iteration and corresponding values					
		0	1	2	3	4	5
I	*e*	2.4726E−1	9.0403E−2	5.4826E−2	8.0814E−3	8.0782E−3	8.0774E−3
	$e_h$	–	2.8013E−2	3.6179E−3	7.2392E−4	1.5669E−4	3.6362E−4
	$q_h$	–	–	77.4294	4.9977	4.6200	4.3092
II	*e*	8.6794E−2	4.0215E−3	3.1259E−5	2.9243E−5	2.9104E−5	2.9100E−5
	$e_h$	–	1.0366E−4	3.4624E−6	3.1645E−7	2.8591E−7	2.8503E−7
	$q_h$	–	–	29.9437	10.9409	1.1068	1.0031
III	*e*	1.6827E−3	9.2546E−4	7.4972E−5	7.4802E−5	7.4792E−5	7.4753E−5
	$e_h$	–	9.2858E−4	7.6389E−5	6.6424E−6	5.9675E−6	5.5203E−6
	$q_h$	–	–	12.1564	11.5004	1.1131	1.0817

**Table 2:** The relation between convergence rate and overlapping degree (Mesh II)

$\mu _1$	$\mu _2$	*k*	Number of iteration and corresponding values					
			0	1	2	3	4	5
1.5	1.2	*e*	6.4728E−2	4.6532E−3	3.4571E−5	2.6119E−5	2.6084E−5	2.6002E−5
		$e_h$	–	2.0222E−3	1.2045E−4	4.5076E−5	9.0874E−6	9.0244E−6
		$q_h$	–	–	16.7890	3.8033	4.9290	1.0660
1.5	1.0	*e*	4.5186E−2	1.0521E−3	9.0705E−5	5.4413E−5	1.2218E−5	1.2103E−5
		$e_h$	–	1.3736E−3	4.8967E−5	2.6640E−7	1.4184E−7	7.5349E−7
		$q_h$	–	–	28.0516	18.3810	2.7813	2.8248
1.5	0.8	*e*	1.4825E−3	6.7734E−4	9.2125E−5	1.8249E−5	5.6719E−6	5.5017E−6
		$e_h$	–	6.4936E−4	2.1429E−5	1.2093E−6	8.2674E−8	1.0827E−8
		$q_h$	–	–	30.3022	17.7197	14.62807	7.6359

From Table 1, we can see the convergence is really fast. Both *e* and $e_h$ are smaller than them on former mesh. And the Fig. 2 shows us the errors converge rapidly. Both of them reveal that the fine the mesh, the faster the convergence. The numbers of Table 2 testify the remark in “The error estimates of the algorithm” section. By taking different $\mu _1$ and $\mu _2$, we chose 3 couples of artificial boundaries. Geometrically, the bigger the $|\mu _1-\mu _2|$, the bigger the overlapping domain. Within the same triangular partition (Mesh II), we conclude that the bigger the overlapping domain, the faster the convergence.

### *Example 2*

Generally, the $\Omega$ is chosen as a prolate ellipsoidal. Set the semi-interfocal $f_0=4$ and $\Gamma _0=\{(\mu ,\theta ,\varphi )| \mu _0=0.5,\theta \in [0,\pi ],\varphi \in [0,2\pi ]\}$. Set $K_1= K_2=1$. Thus, the exact solution of problem (5) is

$$ u=\frac{1}{((x^2+y^2)/ K_1+ z^2/ K_2)^{1/2}}.$$

and $$g=u|_{\Gamma _0}$$.

**Fig. 3:**
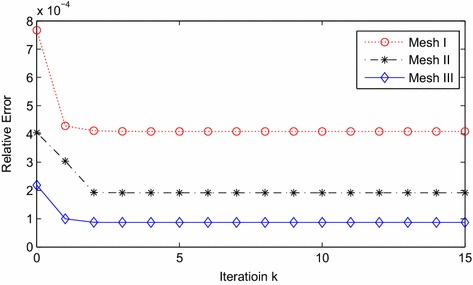
Maximal errors in relative maximum norm

Similarly, we choose two artificial boundaries $\Gamma _1$ and $\Gamma _2$, which are both confocal with $\Gamma _0=\partial {\Omega }$ as $f_1=f_2=f_0=6$. Let $\Gamma _1=\{(\mu ,\theta ,\varphi )| \mu _1=2.5,\theta \in [0,\pi ],\varphi \in [0,2\pi ]\}$ and $\Gamma _2=\{(\mu ,\theta ,\varphi )| \mu _2=2.0,\theta \in [0,\pi ],\varphi \in [0,2\pi ]\}$. The corresponding results are the case in Tables 3, 4 and Fig. 3.

**Table 3:** The relation between convergence rate and mesh: $\mu _1=2.5$, $\mu _2=2.0$

Mesh	k	Number of iteration and corresponding values					
		0	1	2	3	4	5
I	*e*	2.1078E−2	8.4562E−3	5.9623E−3	4.6782E−3	4.6511E−3	4.6407E−3
	$e_h$		9.0022E−4	3.0713E−5	2.1630E−6	1.5593E−6	1.1858E−6
	$q_h$			29.3106	14.1992	1.3871	1.3150
II	*e*	8.3741E−3	7.6501E−3	4.6829E−3	9.4296E−4	8.6241E−4	8.5788E−4
	$e_h$	–	7.7637E−4	1.4383E−6	3.7605E−8	9.6070E−9	2.4529E−9
	$q_h$	–	–	53.9787	38.2471	3.9143	3.9166
III	*e*	1.8257E−3	5.4865E−4	4.2731E−5	3.5722E−5	3.5605E−5	3.5592E−5
	$e_h$	–	1.0350E−6	5.2502E−9	1.2387E−10	3.6938E−11	5.0933E−11
	$q_h$	–	–	197.1280	51.8669	11.4751	6.2403

**Table 4:** The relation between convergence rate and overlapping degree (Mesh II)

$\mu _1$	$\mu _2$	*k*	Number of iteration and corresponding values					
			0	1	2	3	4	5
2.5	1.8	*e*	7.4537E−3	8.6547E−4	4.6829E−4	9.5781E−5	8.7710E−5	8.7058E−5
		$e_h$	–	6.0775E−7	4.7353E−8	5.3837E−9	6.2859E−10	5.6858E−10
		$q_h$	–	–	12.8344	8.7955	8.5647	1.1055
2.5	1.6	*e*	2.4832E−3	7.6489E−4	5.4952E−5	3.6848E−5	2.6981E−5	2.6773E−5
		$e_h$	–	2.9321E−7	1.1713E−8	5.8642E−10	2.8518E−10	2.1763E−10
		$q_h$	–	–	25.0324	19.9742	2.0563	1.3104
2.5	1.4	*e*	5.4377E−4	7.6811E−5	6.8129E−6	8.1056E−7	8.0859E−7	8.05378E−7
		$e_h$	–	4.2367E−7	6.0310E−9	1.0814E−10	1.9075E−11	9.2494E−12
		$q_h$	–	–	70.2475	55.76912	5.6694	2.06226

The data of Tables 3 and 4 show us a good convergence. And the analysis of the numbers can be similar to Example 1.

## Conclusions

In this paper, we construct a Schwarz alternating algorithm for the anisotropic problem on the unbounded domain. The algorithm uses the DDM based on FEM and natural boundary element method. The theoretical analysis shows its convergence is first-order. Further, the rate of convergence is dependent on the overlapping domain. Some numerical examples testify the theoretical conclusions. We can investigate the Schwarz alternating algorithm for anisotropic problem with three different parameters over unbounded domain. Full details and results will be given in a future publication.
